# Intercropping Enhances Productivity and Maintains the Most Soil Fertility Properties Relative to Sole Cropping

**DOI:** 10.1371/journal.pone.0113984

**Published:** 2014-12-08

**Authors:** Zhi-Gang Wang, Xin Jin, Xing-Guo Bao, Xiao-Fei Li, Jian-Hua Zhao, Jian-Hao Sun, Peter Christie, Long Li

**Affiliations:** 1 Key Laboratory of Plant and Soil Interactions, Ministry of Education, College of Resources and Environmental Sciences, China Agricultural University, Beijing 100193, China; 2 Institute of Soils, Fertilizers and Water-Saving Agriculture, Gansu Academy of Agricultural Sciences, Lanzhou 730070, China; DOE Pacific Northwest National Laboratory, United States of America

## Abstract

Yield and nutrient acquisition advantages are frequently found in intercropping systems. However, there are few published reports on soil fertility in intercropping relative to monocultures. A field experiment was therefore established in 2009 in Gansu province, northwest China. The treatments comprised maize/faba bean, maize/soybean, maize/chickpea and maize/turnip intercropping, and their correspoding monocropping. In 2011 (the 3^rd^ year) and 2012 (the 4^th^ year) the yields and some soil chemical properties and enzyme activities were examined after all crop species were harvested or at later growth stages. Both grain yields and nutrient acquisition were significantly greater in all four intercropping systems than corresponding monocropping over two years. Generally, soil organic matter (OM) did not differ significantly from monocropping but did increase in maize/chickpea in 2012 and maize/turnip in both years. Soil total N (TN) did not differ between intercropping and monocropping in either year with the sole exception of maize/faba bean intercropping receiving 80 kg P ha^−1^ in 2011. Intercropping significantly reduced soil Olsen-P only in 2012, soil exchangeable K in both years, soil cation exchangeable capacity (CEC) in 2012, and soil pH in 2012. In the majority of cases soil enzyme activities did not differ across all the cropping systems at different P application rates compared to monocrops, with the exception of soil acid phosphatase activity which was higher in maize/legume intercropping than in the corresponding monocrops at 40 kg ha^−1^ P in 2011. P fertilization can alleviate the decline in soil Olsen-P and in soil CEC to some extent. In summary, intercropping enhanced productivity and maintained the majority of soil fertility properties for at least three to four years, especially at suitable P application rates. The results indicate that maize-based intercropping may be an efficient cropping system for sustainable agriculture with carefully managed fertilizer inputs.

## Introduction

Intercropping, the growth of two or more crop species simultaneously (co-growth) in the same field area, has been widely practiced worldwide [1], [2]. Developing countries such as China, India, Indonesia, Niger, Mali and Ethiopia have shown considerable interest in intercropping to enhance productivity [3], [4]. In particular, cereal/legume intercropping is commonly employed in China and sub-Saharan Africa and has shown overyielding and nutrient acquisition advantages under adverse conditions [1], [5], [6], [7], [8], [9]. Furthermore, intercropping also provides an important pathway to reduce soil erosion, fix atmospheric N_2_, lower the risk of crop failure or disease and increase land use efficiency [10], [11], [12], [13], [14], [15].

There are many kinds of intercropping in northwest China including wheat/maize, wheat/soybean [16], [17], maize/faba bean [6], [18], [19], maize/potato, wheat/sunflower, wheat/vegetable and maize/vegetable systems [10]. In other regions of the world such as tropical areas of Indonesia, India, Niger and Mali there are jungle rubber-based agroforestry and multistrata agroforestry systems [1], [2], [4], [10]. In Europe there are a number of intercropping systems which include barley (*Hordeum sativum* L.)/pea (*Pisum sativum* L.), wheat/pea and maize/melon intercropping in Denmark, the UK, France, Italy and Germany [10]. In the Americas pea/barley or oats and in Canada wheat/canola or pea and broccoli intercropped with peas, beans, potatoes, oats or cabbage are grown and in the USA maize and soybean are intercropped [10]. Most importantly, intercropping significantly enhances crop yields, for example maize/faba bean intercropping produces 21–23 and 6.5–11.8% yield increases over the corresponding monocultures [6], [19], [20].

There are very close relationships between yield advantage and nutrient acquisition in intercropping systems [12], [21]. Intercropping is an efficient cropping system in terms of resource utilization [1]. For instance, crops with different traits can explore various organic P sources in P-deficient soils [22], [23], [24]. Cereal/legume mixtures can achieve higher P uptake on such soils than the corresponding monocultures in either pot or field conditions [20], [25], [26], [27]. Underlying mechanisms include increased P mobilization via chelation of Ca^2+^ by citrate exuded from the roots of one of the intercropped species [20], [28]. For example, total P acquisition by aboveground parts in intercropped maize and faba bean was significantly higher than in maize or faba bean monoculture in a reclaimed desert soil[18]. Similar results were observed in maize/faba bean, maize/chickpea, maize/soybean and maize/ternip intercropping in a relatively fertile soil [19]. As a result, wheat/maize, wheat/soybean or maize/faba bean intercropping enhanced N acquisition significantly compared with monoculture [16], [17], [29]. Similar results with N acquisition have also been found in wheat/bean intercropping in Africa and in pea/barley intercropping in Denmark [30], [31].

However, little attention has been paid to soil chemical properties and enzyme activities under continuous overyielding and greater nutrient removal from soil by intercropping systems compared with monocultures over a time scale of several years. In general, soil fertility plays an important role in the sustainability of agricultural and natural ecosystems [32], [33], [34], [35]. Soil fertility is defined as the capacity to sustain plant productivity and maintain or enhance water and air quality [36]. Numerous previous studies have investigated soil fertility in relation to fertilization, irrigation, tillage and other factors [37]. To our knowledge, there are few published studies available on intercropping at different P rates on soil chemical properties and enzyme activities over periods of several years. We hypothesize that intercropping can maintain soil fertility by increasing below-ground biomass inputs derived from above-ground biomass overyielding as well as below-ground biodiversity resulting from above-ground crop diversity.

The objective of the present study was therefore to test this hypothesis by investigating whether continuous intercropping continues to maintain overyielding and nutrient removal advantage and to maintain the soil chemical properties and enzyme activities at different fertilizer P application rates.

## Materials and Methods

### Site description

The study was conducted at the Experimental Station of the Institute of Soils, Fertilizers and Water-Saving Agriculture, Gansu Academy of Agricultural Sciences, at Baiyun (38°37′N, 102°40′E), 15 km north of Wuwei city in Gansu Province, northwest China. The site is at an altitude of 1504 m above sea level and the area has a typical arid climate. Annual precipitation and potential evaporation are 150 mm and 2021 mm, respectively. Most of the precipitation occurs between May and October with a unimodal distribution pattern. Annual mean temperature is 7.7°C. Cumulative temperatures ≥0°C and ≥10°C are 3646°C and 3149°C, respectively. Total solar radiation is 5988 MJ m^−2^ year^−1^ and the annual frost-free period is 170–180 days.

The soil type is classified as an Orthic Antrosols [38] with 57% sand and 39% clay. The top 20 cm of the soil profile has a bulk density of 1.40 g cm^−3^. Topsoil physico-chemical properties before the experiment started in 2009 were: OM 19.1 g kg^−1^, total N 1.08 g kg^−1^, Olsen P 20.3 mg kg^−1^, exchangeable K 233 mg kg^−1^, and pH value (2.5∶1 w/v in water) 8.00 [19].

### Experimental design and crop management

The experiment was a two factorial design with three replicates. The main factorial treatments comprised three P application rates (0, 40 and 80 kg P ha^−1^ applied as triple superphosphate) and the sub factorial treatments were maize (*Zea mays* L. cv. Zhengdan no. 958) intercropped with four species, namely faba bean (*Vicia faba* L. cv. Lincan no. 5), soybean (*Glycine max* L. cv. Wuke no. 2), chickpea (*Cicer arietinum* L. cv. Longying no. 1), and turnip (*Brassica campestris* L. cv. Gannan no. 4) and the corresponding five monocultures (maize, faba bean, soybean, chickpea and turnip).

Crop rows for all intercrops and monocultures were oriented in an east-west direction. Each individual plot had an area of 4.0×5.5 m^2^ for monoculture maize, faba bean, soybean, chickpea and turnip, and 5.6×5.5 m^2^ of for the intercropping treatments. There were 4 strips with a width of 1.4 m and 2 rows of maize alternating with 3 rows of legume species or turnip planted in each strip for each intercropping plot. The inter-row distance was 40 cm for maize and 20 cm for the legume species and turnip and was identical in both monocultures and intercrops. The intercropping treatments had a 30-cm-wide gap between maize and associated crop rows. Theoretically, the row design of the intercropping resulted in the two maize rows occupying 80 cm width of the strip and the three associated crop rows or turnip occupying 60 cm width. Consequently, the maize rows occupied 80/140 = 57% of the intercropping area and the companion crop rows occupied 60/140 = 43%. The inter-plant distance was 26 cm for monoculture and intercropped maize and 20 cm for monoculture and intercropped legume species, and turnip was planted by broadcast sowing in each row. The spacing design allowed to comparison between intercropping and monoculture at the same density of maize or associated crops in the intercrops and the monocultures.

All legume species and turnip received the same rate of N (as urea), namely 112.5 kg ha^−1^ and this accounted for half the total amount of fertilizer N applied. The other half of the N application was given to maize and without K fertilizer or farmyard manure was applied to all crops. All the fertilizer P and 112.5 kg N ha^−1^ were evenly broadcast and incorporated into the top 20 cm of the soil profile before sowing. The other half of the fertilizer N (112.5 kg ha^−1^) for maize was applied at two irrigation events at maize stem elongation and pre-tasseling stages. During the growing season all plots were irrigated 6 or 7 times and weeded manually. No fungicides were applied in either year.

### Soil sampling and analysis

Soil samples were collected to 20 cm depth using an auger after harvest to minimize damage to the plots in 2011 and 2012. Three replicate cores were collected from intercropping strips of maize rows and associated crops and mixed together to give composite samples and the same method was used in the monoculture plots. The composite samples were stored in plastic bags, air-dried and sieved through a 2.0 mm mesh and plant residues and roots were removed by hand prior to chemical analysis. Soil samples were also collected and stored at 4°C prior to analysis for soil enzyme activities.

Soil OM was determined by wet oxidation using the acidified dichromate method [39]. Soil total N was measured after Kjeldahl digestion according to standard protocols (SKD-800, Shanghai, Peiou Corporation). Soil Olsen P was determined using standard procedures [40] by colorimetry (Uvmini-1240, Shimadzu Corporation). Soil exchangeable K was extracted using 1 mol L^−1^ ammonium acetate solution buffered at pH 7 and determined by flame photometry (M410, Sherwood Corporation, UK). Soil CEC was measured by the sodium saturation method [41]. Soil pH was measured in soil suspension with deionized-distilled water (2.5∶1, w/v) (pHS-3C, SPSIC Corporation).

Soil urease activity was determined by the method described by Guan (1986) [42] with minor modification. Five grams of fresh soil were placed in a 50-mL volumetric flask together with 1.0 mL toluene. Fifteen minutes later, 5 mL 10% urea solution and 10 mL citrate buffer (pH 6.7) were added. The flask was shaken and then placed in an incubator at 37±0.1°C for 24 h. After incubation, deionized water at 38°C was added to a volume of 50 mL. The suspension was filtered. To a 50-mL volumetric flask were added 1.0 mL of the filtrate with 9 mL deionized water, 4 mL sodium phenate solution and 3 mL sodium hypochlorite solution. Twenty minutes later, deionized water was added up to a volume of 50 mL. Finally, urease activity was determined colorimetrically at 578 nm and expressed as mg NH_3_-N (g soil)^−1^ (24 h)^−1^.

Soil nitrate reductase activity was determined by a colorimetric method [42] with modification. Triplicate 5 g soil samples were incubated with 4 ml of 2,4-dinitrophenol solution 1 ml potassium nitrate solution and 5 ml distilled water at 25°C for 24 h. A similar set up was prepared for the control. The control sample was incubated at −20°C for 24 h. After incubation, 10 ml 4 M KCl solution was added to all the soil samples including the control. This was shaken for 30 minutes and filtered. To 5 ml of the filtrate, 3 ml of NH_4_Cl buffer (pH 8.5) and 2 ml of color reagent were added. This was kept for 15 minutes for color development. Optical density was was determined in a spectrophotometer against the blank at 520 nm. The enzyme activity was expressed as micrograms of NO^−^_2_–N per gram daily

Soil sucrase activity was determined by the method described by Guan (1986) with modification. Five grams of fresh soil were placed in a 50-mL Erlenmeyer flask together with 15 mL 8% of sucrose solution, 5 mL phosphate buffer (pH 5.5) and 1 ml of toluene. The flask was shaken and then placed in an incubator at 37.0±0.1°C for 24 h. After incubation, the sample was filtered through a quantitative filter paper. Then, 1 mL of the filtrate and 3 mL salicylic acid were taken to a 50-mL volumetric flask and heated for 5 min at 100°C in a water bath. After heating, the flask was cooled for 3 min with flowing tap water and deionized water was added to make up to 50 mL, and sucrase activity was measured colorimetrically at 508 nm (U-2800, Japan). Sucrase activity is expressed as mg glucose g soil^−1^ (24 h)^−1^.

Soil acid phosphatase activity was determined using p-nitrophenyl phosphate disodium (PNPP) as substrate [43]. On the basis of a modified universal buffer stock solution, the pH for the acid phosphatase analysis was adjusted to 6.5 with HCl. The pNP released by phosphatase was determined colorimetrically at 400 nm. Enzyme activity was expressed as micrograms of p-nitrophenol produced per gram of soil.

### Plant sampling and analysis

At maturity, two continuous rows of maize and three continuous rows of associated crops as a strip were collected from both the intercropping and monoculture treatments and the grain yields and above-ground dry matter yields were calculated on a per hectare basis.

Samples of aboveground parts were divided into grain and straw at maturity. Plant materials were over-dried at 70°C for 48 h and ground. Nitrogen (N), phosphorus (P) and potassium (K) concentrations in grain and straw were determined on ground sub-samples of oven-dried plant material after digestion in a mixture of concentrated H_2_SO_4_ and H_2_O_2_. Nitrogen was measured by the micro-Kjeldahl procedure with 5 ml digestion solution, P by the vanadomolydate method, and K by flame photometry. Aboveground nutrient acquisition (N, P and K) of each crop species was calculated as the sum of grain and straw nutrient acquisition which was determined by the product of nutrient concentration and grain or straw yields based on the land area occupied by the crops.

### Calculations

In order to compare grain yield and nutrient acquisition, soil chemical properties and enzyme activities in faba bean/maize, soybean/maize, chickpea/maize and turnip/maize intercropping were compared with the corresponding monocultures. In the field experiment intercropping comprised two crops and the properties determined in intercropping were compared with the weighted means of the two monoculture crops based on their proportions in the intercropping system.

In the present study we calculated the weighted means of grain yields by monoculture crop species as follows:
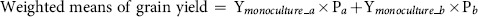
(1)


Where Y*_monoculture_a_* and Y*_monoculture_b_* are the grain yields of crops *a* and *b* in the monoculture (Mono). P*_a_* and P*_b_* are the proportions of the area occupied by the individual crops in the intercropping system (Inter). P*_a_* is determined by P*_a_* = W*_a_*/(W*_a_*+W*_b_*) and P*_b_* = W*_b_*/(W*_a_*+W*_b_*), where W*_a_* and W*_b_* are the widths of crops *a* and *b* in intercropping strips. The above formulas were also used to calculate the nutrient acquisition, soil chemical properties and enzyme activities by the monocultures.

### Statistical analysis

The experimental data were subjected to analysis of variance using either two factorial (P application rate and cropping system) analysis (i.e. above-ground biomass, N, P and K acquisition of aboveground parts) or three factorial (year, P application rate and cropping system) analysis (i.e. grain yields, all soil properties) including the interactions between factors involved using the SAS software package (SAS Institute, 2003, version 8.2). The mean values (n = 3) were compared by LSD (least significant difference) at the 5% level.

## Results

### Crop productivity

**Figure 1:**
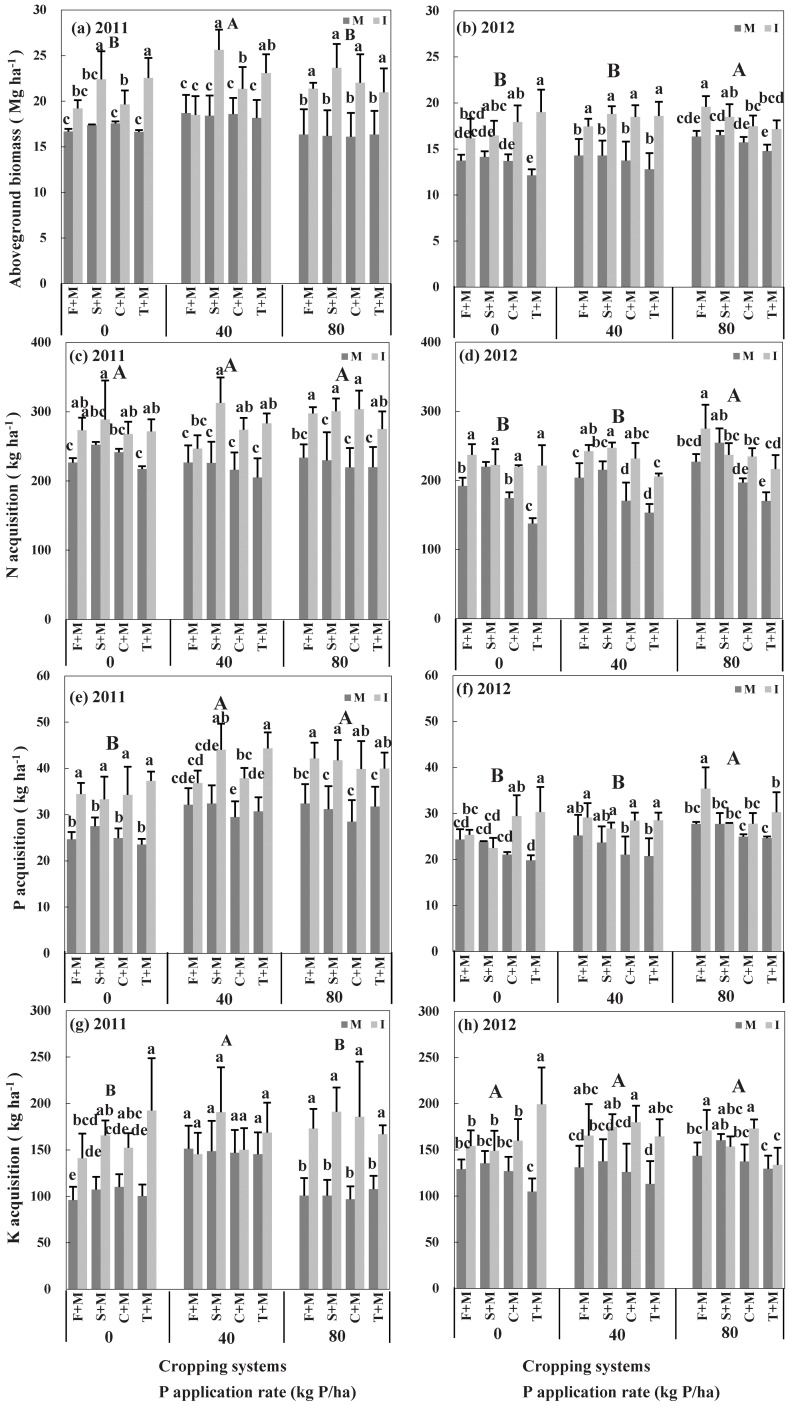
Aboveground biomass and N, P and K acquisition of intercropping and weighted means of monocropped crops in 2011 and 2012. Different capital letters indicate significant differences at *P*<0.05 by LSD among different P application rates; different lowercase letters with the same P application rates indicate significant differences at *P*<0.05 by LSD among different cropping systems; F+M, S+M, C+M and T+M indicate intercropping of maize with faba bean, maize and soybean, and chickpea and turnip, respectively; M and I indicate monoculture and intercropping, respectively. The error bars are standard deviation with 3 replicates.

Generally, across all four crop combinations, regardless of intercropping or monocropping, both the grain yields and aboveground biomass were highest at 40 kg P ha^−1^ in 2011 but at 80 kg P ha^−1^ in 2012 (Fig. 1). Grain yields did not differ significantly with P application in 2011 but were higher at 80 kg P ha^−1^ in 2012 (Table 1).

**Table 1:** Grain yield (Mg ha^−1^) as affected by main effects of P application and subplot effects of cropping system in 2011 and 2012.

Year	P rate (kg ha^−1^)	Grain yield of intercropped and weighted means of corresponding monocropped crops (Mg ha^−1^)										
		Maize + faba bean		Maize + soybean		Maize + chickpea		Maize + turnip		Average		
		Mono	Inter	Mono	Inter	Mono	Inter	Mono	Inter	Mono	Inter	Mean
2011	0	8.5c	10.9b	8.7c	11.2ab	9.0c	11.5ab	8.3c	12.1a	8.6b	11.4a	10.0A
	40	9.7c	10.8bc	9.7c	14.2a	9.9c	12.7ab	9.3c	12.9ab	9.6b	12.6a	11.1A
	80	9.1b	11.3a	8.9b	12.5a	9.1b	12.9a	8.4b	11.5a	8.9b	12.2a	10.5A
	**Mean**	**9.1C**	**11.1B**	**9.1C**	**12.6A**	**9.3C**	**12.4A**	**8.7C**	**12.1AB**	**9.1B**	**12.1A**	**10.6**
2012	0	6.2cd	7.3bc	6.7bcd	7.7abc	6.5bcd	9.1a	5.5d	7.9ab	6.2b	8.0a	7.1B
	40	6.7c	8.3ab	6.9bc	8.9a	6.8c	8.8a	5.8c	8.4ab	6.6b	8.7a	7.6AB
	80	7.9bc	9.3a	8.2abc	8.4ab	7.9bc	7.9bc	6.9c	8.1abc	7.7a	8.4a	8.1A
	**Mean**	**6.9B**	**8.3A**	**7.3B**	**8.3A**	**7.0B**	**8.6A**	**6.1C**	**8.1A**	**6.8B**	**8.3A**	**7.6**
ANOVA												
	Year (Y)				<0.001						<0.001	
	P rate (P)				<0.001						0.020	
	Cropping system (C)				0.011						<0.001	
	Y×P				<0.001						0.209	
	Y×C				0.230						0.004	
	P×C				<0.001						0.640	
	Y×P×C				0.216						0.296	

Values are means of three replicates. Values followed by the same lowercase letters are not significantly different among different cropping systems with the same P rate in one year at the 5% level by LSD (horizonal comparison); values followed by the same capital letters are not significantly different among different P rates (vertical comparison) or among different cropping systems (horizonal comparison) in one year at the 5% level by LSD. Values under ANOVA are the probabilities (*P* values) of the sources of variation.

Aboveground biomass was significantly (P<0.05) enhanced in faba bean/maize intercropping receiving 40 kg P ha^−1^ in 2012 and 80 kg P ha^−1^ in both years, in soybean/maize at all P application rates and years with the sole exception of 2012, in chickpea/maize receiving 80 kg P ha^−1^ in 2011 and all three P application rates in 2012, and turnip/maize intercropping receiving all three P rates in both years (Fig 1a and 1b). Similarly, grain yields significantly (P<0.05) overyielded in all four intercropping systems receiving all three P application rates in both 2011 and 2012 with the sole exception of faba bean/maize receiving 40 kg P ha^−1^ in 2011 and faba bean/maize and soybean/maize intercropping without P application, and soybean/maize, chickpea/maize, turnip/maize intercropping receiving 80 kg P ha^−1^ in 2012 (Table 1).

### Nutrient acquisition

P applications did not significantly increase N acquisition of aboveground parts regardless of the four cropping systems in 2011 but 80 kg P ha^−1^ increased N uptake by 3.0 and 11.3% compared with zero P or 40 kg P ha^−1^ in 2012 (Fig 1c and 1d).

Similar to aboveground biomass, all four intercropping systems receiving all three P application rates removed significantly more N than did their corresponding monocultures in both years, with the exception of faba bean/maize intercropping receiving 40 kg P ha^−1^, chickpea/maize intercropping without P application in 2011 and soybean/maize intercropping receiving 0 and 80 kg P ha^−1^ (Fig 1c and 1d).

Average aboveground P acquisition across all crop combinations increased significantly (P<0.05) at 40 and 80 kg P ha^−1^ in 2011 but only at 80 kg P ha^−1^ in 2012 (Fig 1e and 1f).

All four intercropping systems removed significantly (P<0.05) greater P from soil than the weighted means of corresponding monocultures, with exception of faba bean/maize and chickpea/maize intercropping received 40 kg P ha^−1^ in 2011, and faba bean/maize and soybean/maize intercropping received 0 and 40 kg P ha^−1^ in 2012 and soybean/maize and chickpea/maize intercropping received 80 kg P ha^−1^ in 2012 (Fig. 1f). Potassium acquisiton of intercropping showed a similar trend to P acquisition (Fig 1g and h).

### Soil chemical properties

Soil OM over all four crop systems was enhanced significantly at 80 kg P ha^−1^ in comparison with 40 kg P ha^−1^ in 2011 but not in 2012 (Table 2). Averaged over the three P levels, soil OM was significantly (P<0.05) higher by 7.4% and 5.4% in maize/turnip intercropping in both years and by 9.8% in maize/chickpea intercropping in 2012 compared to the weighted means of the corresponding monocultures (Table 2). In contrast, soil OM declined significantly (P<0.05) by 6.8% and 6.0% in maize/faba bean and maize/soybean intercropping than the corresponding monocultures but only in 2012 (Table 2). For individual P levels, soil OM was enhanced by 13.8% and 9.6% in maize/turnip intercropping receiving only 40 kg P ha^−1^ in both years and by 19.9% in maize/chickpea intercropping receiving 40 kg P ha^−1^ in 2012 and by 5.7% in maize/soybean intercropping without P application in 2011 (Table 2).

**Table 2:** Soil OM (g kg^−1^) content as affected by main effects of P application and subplot effects of cropping system in 2011 and 2012.

Year	P rate (kg ha^−1^)	Soil organic matter content of intercropped and weighted means of corresponding monocropped crops (g kg^−1^)										
		Maize + faba bean		Maize + soybean		Maize + chickpea		Maize + turnip		Average		
		Mono	Inter	Mono	Inter	Mono	Inter	Mono	Inter	Mono	Inter	Mean
2011	0	21.9abcd	21.2d	21.2d	22.4abc	22.7a	21.4cd	21.6bcd	22.5ab	21.9a	21.9a	21.9AB
	40	21.7b	20.5b	21.2b	20.8b	21.3b	21.6b	21.0b	23.9a	21.3a	21.7a	21.5B
	80	22.1ab	22.1ab	22.5ab	21.8b	22.1ab	22.3ab	22.2ab	23.3a	22.2a	22.4a	22.3A
	**Mean**	**21.9BC**	**21.2C**	**21.6BC**	**21.6BC**	**22.0B**	**21.8BC**	**21.6BC**	**23.2A**	**21.8A**	**22.0A**	**21.9**
2012	0	21.2b	20.5b	21.3b	21.0b	22.3ab	24.6a	22.0ab	24.3a	21.7a	22.6a	22.2A
	40	22.3c	20.3e	20.9cde	20.6de	22.1cd	26.5a	21.8cde	23.9b	21.8a	22.8a	22.3A
	80	23.0a	21.3ab	23.0a	19.7b	23.2a	23.1a	23.1a	22.3a	23.1a	21.6a	22.3A
	**Mean**	**22.2C**	**20.7DE**	**21.7CD**	**20.4E**	**22.5BC**	**24.7A**	**22.3C**	**23.5B**	**22.2A**	**22.3A**	**22.3**
ANOVA												
	Year (Y)				0.020						0.082	
	P rate (P)				0.118						0.299	
	Cropping system (C)				<0.001						0.427	
	Y×P				0.153						0.342	
	Y×C				<0.001						0.943	
	P×C				0.003						0.034	
	Y×P×C				0.056						0.041	

Values are means of three replicates. Values followed by the same lowercase letters are not significantly different among different cropping systems with the same P rate in one year at the 5% level by LSD (horizonal comparison); values followed by the same capital letters are not significantly different among different P rates (vertical comparison) or among different cropping systems (horizonal comparison) in one year at the 5% level by LSD. Values under ANOVA are the probabilities (*P* values) of the sources of variation.

Similarly, soil total N was little affected by P application rate or cropping system in both years. Interestingly, soil total N was not changed by intercropping or monoculture across both years with the exception of maize/faba bean intercropping by 4.0% in 2012 over all P rates. Soil total N in maize/faba bean intercropping was enhanced by 8.3% when receiving 80 kg P ha^−1^ in 2011 (Table S1).

In general, soil Olsen P increased with increasing fertilizer P application rate and significant differences were observed between 0 and 40 kg ha^−1^ P in both 2011 and 2012 averaged over all cropping systems but further P application (80 kg P ha^−1^) showed no response to the significant increase in soil Olsen P (Table 3). In 2011 there were no significant differences in soil Olsen P between intercropping and the weighted means of the corresponding monocrops at individual P levels or the average over all P rates (Table 4). In 2012, however, the differences became significant, especially under zero P and soil Olsen P in maize/faba bean and maize/soybean intercropping was significantly reduced by 32.5% and 46.8% compared with the corresponding monocrops. At 80 kg P ha^−1^, soil Olsen P decreased significantly with maize/faba bean, maize/soybean, maize/chickpea and maize/turnip intercropping and by 32.5%, 28.6%, 21.8% and 28.6%, respectively (Table 3). Similar results were observed in maize/faba bean, and maize/soybean maize/turnip intercropping with the exception of the maize/chickpea combination (Table 3).

**Table 3:** Soil Olsen P (mg kg^−1^) as affected by main effects of P application and subplot effects of cropping system in 2011 and 2012.

Year	P rate (kg ha^−1^)	Soil Olsen P of intercropped and weighted means of corresponding monocropped crops (mg kg^−1^)										
		Maize + faba bean		Maize + soybean		Maize + chickpea		Maize + turnip		Average		
		Mono	Inter	Mono	Inter	Mono	Inter	Mono	Inter	Mono	Inter	Mean
2011	0	18.7ab	12.3bc	15.7abc	11.5c	16.3abc	22.6a	17.1abc	19.8a	17.0a	16.5a	16.8B
	40	26.9ab	23.5ab	23.0ab	34.7a	24.3ab	22.4b	29.6ab	18.0b	26.0a	24.7a	25.3A
	80	30.2a	26.4a	29.7a	27.0a	33.5a	38.6a	35.4a	34.1a	32.2a	31.5a	31.8A
	**Mean**	**25.3AB**	**20.8B**	**22.8AB**	**24.4AB**	**24.7AB**	**27.9A**	**27.4A**	**23.9AB**	**25.0A**	**24.2A**	**24.6**
2012	0	24.0a	16.2b	26.7a	14.2b	27.6a	30.1a	24.3a	28.3a	25.6a	22.2a	23.9B
	40	34.7ab	25.5bc	33.4abc	29.2abc	33.8abc	38.1a	33.6abc	23.8c	33.9a	29.2a	31.5A
	80	39.1ab	26.4d	37.1abc	26.5d	42.6a	33.3bcd	41.3ab	29.5cd	40.0a	28.9b	34.5A
	**Mean**	**32.6A**	**22.7B**	**32.4A**	**23.3B**	**34.6A**	**33.9A**	**33.1A**	**27.2B**	**33.2A**	**26.8B**	**27.0**
ANOVA												
	Year (Y)				<0.001						0.001	
	P rate (P)				0.083						<0.001	
	Cropping system (C)				0.000						0.016	
	Y×P				0.029						0.385	
	Y×C				0.441						0.055	
	P×C				<0.001						0.494	
	Y×P×C				0.337						0.479	

Values are means of three replicates. Values followed by the same lowercase letters are not significantly different among different cropping systems with the same P rate in one year at the 5% level by LSD (horizonal comparison); values followed by the same capital letters are not significantly different among different P rates (vertical comparison) or among different cropping systems (horizonal comparison) in one year at the 5% level by LSD. Values under ANOVA are the probabilities (*P* values) of the sources of variation.

**Table 4:** Soil exchangeable K (mg kg^−1^) as affected by main effects of P application and subplot effects of cropping system in 2011 and 2012.

Year	P rate (kg ha^−1^)	Soil exchangeable K of intercropped and weighted means of corresponding monocropped crops (mg kg^−1^)										
		Maize + faba bean		Maize + soybean		Maize + chickpea		Maize + turnip		Average		
		Mono	Inter	Mono	Inter	Mono	Inter	Mono	Inter	Mono	Inter	Mean
2011	0	95.9a	69.9c	98.6a	79.0b	95.7a	72.8bc	96.7a	74.7bc	96.7a	74.1b	85.4A
	40	91.6a	66.8d	95.3a	78.4bc	96.4a	80.3b	96.6a	74.3c	95.0a	75.0b	85.0A
	80	96.3a	71.4c	93.6a	80.3b	97.6a	80.7b	99.5a	75.4bc	96.8a	77.0b	86.9A
	**Mean**	**94.6A**	**69.4D**	**95.8A**	**79.2B**	**96.6A**	**77.9BC**	**97.6A**	**74.8C**	**96.2A**	**75.4B**	**85.7**
2012	0	83.9a	70.5b	75.6ab	76.5ab	80.3ab	82.7a	84.6a	84.6a	81.1a	78.6a	79.8A
	40	87.6ab	78.8bcd	74.3d	84.9abc	77.3cd	77.4cd	87.9a	84.9abc	81.0a	81.5a	81.3A
	80	85.1b	74.2bc	78.5b	80.6b	79.5b	64.9c	99.7a	76.4bc	79.9a	74.0b	76.9A
	**Mean**	**85.5AB**	**74.5E**	**76.1DE**	**80.7BCD**	**79.0CDE**	**75.0DE**	**90.7A**	**82.0BC**	**80.6A**	**78.0B**	**79.3**
ANOVA												
	Year (Y)				<0.001						<0.001	
	P rate (P)				0.722						0.801	
	Cropping system (C)				<0.001						<0.001	
	Y×P				0.204						0.334	
	Y×C				<0.001						<0.001	
	P×C				0.109						0.090	
	Y×P×C				0.003						0.034	

Values are means of three replicates. Values followed by the same lowercase letters are not significantly different among different cropping systems with the same P rate in one year at the 5% level by LSD (horizonal comparison); values followed by the same capital letters are not significantly different among different P rates (vertical comparison) or among different cropping systems (horizonal comparison) in one year at the 5% level by LSD. Values under ANOVA are the probabilities (*P* values) of the sources of variation.

Soil exchangeable K was equally affected by fertilizer P in 2011 and 2012 over all cropping systems (Table 4). Averaged over the three P rates, soil exchangeable K decreased significantly (P<0.05) by 12.9–26.6%, 5.1–19.4% and 9.6–16.6% in maize/faba bean, maize/chickpea and maize/turnip intercropping in comparison with the weighted means of the corresponding monocultures over thetwo years. However, only maize/soybean intercropping decreased soil exchangeable K by 17.3% compared to the corresponding monocrops in 2011 (Table 4). Similar results were also detected in the majority of cases in all four intercropping systems at the individual P levels (Table 4).

Soil CEC over all three P rates was not influenced significantly (P>0.05) by intercropping system in 2011 but was reduced significantly by 5.1%, 9.6% and 7.2% in maize/faba bean, maize/soybean and maize/turnip intercropping in 2012 (with the exception of maize/chickpea intercropping) (Table 5). For the individual P levels, soil CEC was reduced significantly by 27.1% and 24.3% in maize/faba bean and maize/chickpea intercropping receiving 80 kg P ha^−1^ in 2011 and by 6.9%, 14.5%, 8.8%, and 8.9% in maize/faba bean, maize/soybean, maize/chickpea and maize/turnip intercropping systems with zero P and by 10.8%, 11.7%, and 10.0% at 40 kg ha^−1^ P with the exception of maize/chickpea intercropping in 2012 (Table 5).

**Table 5:** Soil CEC (cmol kg^−1^) as affected by main effects of P application and subplot effects of cropping system in 2011 and 2012.

Year	P rate (kg ha^−1^)	Soil CEC of intercropped and weighted means of corresponding monocropped crops (cmol kg^−1^)										
		Maize + faba bean		Maize + soybean		Maize + chickpea		Maize + turnip		Average		
		Mono	Inter	Mono	Inter	Mono	Inter	Mono	Inter	Mono	Inter	Mean
2011	0	21.0a	22.9a	19.6a	23.5a	19.4a	23.7a	20.4a	24.8a	20.1a	23.8a	21.9B
	40	22.9a	22.1a	21.8a	20.1a	23.2a	25.1a	22.5a	22.9a	22.6a	22.6a	22.6B
	80	29.1a	21.2bc	26.6ab	27.2ab	26.8ab	20.3c	26.5ab	23.0abc	27.2a	22.9a	25.1A
	**Mean**	**24.3A**	**22.1A**	**22.7A**	**23.6A**	**23.1A**	**23.0A**	**23.1A**	**23.6A**	**23.3A**	**23.1A**	**23.2**
2012	0	21.8a	20.3bc	22.8a	19.5c	21.5ab	19.6c	21.3ab	19.4c	21.9A	19.7B	20.8B
	40	22.2bc	19.8d	22.2bc	19.6d	22.2bc	23.4ab	24.0a	21.6c	22.6A	21.1B	21.9A
	80	20.4abc	21.2abc	20.5abc	20.2bc	19.8c	21.7a	21.5ab	20.6abc	20.6A	20.9A	20.4B
	**Mean**	**21.5B**	**20.4CD**	**21.9AB**	**19.8D**	**21.2BC**	**21.6AB**	**22.2A**	**20.6C**	**21.7A**	**20.6B**	**21.1**
ANOVA												
	Year (Y)				<0.001						0.003	
	P rate (P)				0.028						0.151	
	Cropping system (C)				0.734						0.292	
	Y×P				0.004						0.055	
	Y×C				0.697						0.499	
	P×C				0.265						0.226	
	Y×P×C				0.021						0.009	

Values are means of three replicates. Values followed by the same lowercase letters are not significantly different among different cropping systems with the same P rate in one year at the 5% level by LSD (horizonal comparison); values followed by the same capital letters are not significantly different among different P rates (vertical comparison) or among different cropping systems (horizonal comparison) in one year at the 5% level by LSD. Values under ANOVA are the probabilities (*P* values) of the sources of variation.

In general, a moderate P rate (40 kg P ha^−1^) did not alter soil pH but a further increase in P application rate (80 kg P ha^−1^) significantly reduced soil pH in comparison with zero P or moderate P over all cropping systems in both 2011 and 2012 (Table S2). There were no differences between intercropping and the corresponding monocultures for the individual P levels but only maize/soybean intercropping enhanced soil pH by 6.2% compared with the corresponding monoculture averaged over all P levels in 2011 but with a significant decreased in maize/soybean, maize/soybean and maize/turnip intercropping compared with the weighted means of corresponding monoculture in 2012. Interestingly, maize/chickpea intercropping showed a slight increase in soil pH ranging from 0.07 to 0.15 pH units in 2012 (Table S2).

### Soil enzyme activities

Soil urease activity averaged over all cropping systems was unaffected by fertilizer P application rate in both 2011 and 2012 (Table 6). Averaged over three P rates, urease activity was not altered by intercropping over the two years with the exception of maize/soybean intercropping in 2011 (Table 6). At the individual P level, soil urease activity in maize/soybean and maize/turnip intercropping with zero P was enhanced by 18.1% and 19.3% and maize/chickpea intercropping receiving 80 kg P ha^−1^ showed a decrease of 28.1% in 2011, and maize/faba bean intercropping with zero P increased by 15.7% in 2012 (Table 6).

**Table 6:** Urease activity as affected by main effects of P application and subplot effects of cropping system in 2011 and 2012.

Year	P rate (kg ha^−1^)	Urease activity of intercropped and weighted means of corresponding monocropped crops (mg NH_4_^+^-N g^−1^ soil d^−1^)										
		Maize + faba bean		Maize + soybean		Maize + chickpea		Maize + turnip		Average		
		Mono	Inter	Mono	Inter	Mono	Inter	Mono	Inter	Mono	Inter	Mean
2011	0	1.204bc	1.417ab	1.275bc	1.506a	1.241bc	1.210bc	1.159c	1.383ab	1.220b	1.379a	1.299A
	40	1.270b	1.231b	1.368ab	1.453ab	1.334ab	1.538a	1.346ab	1.274b	1.330a	1.374a	1.352A
	80	1.227cd	1.161d	1.322bcd	1.460ab	1.612a	1.159d	1.205cd	1.355bc	1.342a	1.284a	1.313A
	**Mean**	**1.234C**	**1.270C**	**1.322BC**	**1.473A**	**1.396AB**	**1.302BC**	**1.237C**	**1.337BC**	**1.297A**	**1.346A**	**1.321**
2012	0	1.832b	2.119a	1.538c	1.554c	1.958ab	1.969ab	1.885ab	2.029ab	1.803a	1.918a	1.861A
	40	1.922ab	1.954ab	1.648c	1.753bc	1.881ab	1.840bc	1.883ab	2.051a	1.834a	1.900a	1.867A
	80	1.822ab	1.878ab	1.629b	1.653b	1.977a	2.020a	2.066a	2.009a	1.874a	1.890a	1.882A
	**Mean**	**1.859B**	**1.984A**	**1.605C**	**1.653C**	**1.939AB**	**1.943AB**	**1.945AB**	**2.030A**	**1.837B**	**1.902A**	**1.870**
ANOVA												
	Year (Y)				<0.001						<0.001	
	P rate (P)				0.007						0.582	
	Cropping system (C)				<0.001						0.020	
	Y×P				0.840						0.575	
	Y×C				<0.001						0.714	
	P×C				0.101						0.034	
	Y×P×C				0.217						0.574	

Values are means of three replicates. Values followed by the same lowercase letters are not significantly different among different cropping systems with the same P rate in one year at the 5% level by LSD (horizonal comparison); values followed by the same capital letters are not significantly different among different P rates (vertical comparison) or among different cropping systems (horizonal comparison) in one year at the 5% level by LSD. Values under ANOVA are the probabilities (*P* values) of the sources of variation.

There were no significant differences in soil acid phosphatase activity between intercropping and the corresponding monocrops over all P rates in 2011 or 2012 except for maize/chickpea intercropping in which activity was enhanced by 39.3% in 2011 (Table 7). At the individual P levels, soil acid phosphatase activity in maize/faba bean, maize/soybean and maize/chickpea intercropping receiving 40 kg P ha^−1^ were 65.0%, 26.2% and 53.2% higher than in the monocrops and maize/turnip intercropping without P increased by 86.4% in 2011. Maize/soybean intercropping receiving 40 kg P ha^−1^ was 52.9% greater than the monocrops in 2012 (Table 7).

**Table 7:** Acid phosphatase activity as affected by main effects of P application and subplot effects of cropping system in 2011 and 2012.

Year	P rate (kg ha^−1^)	Acid phosphatase activity of intercropped and weighted means of corresponding monocropped crops (µg *p*-nitrophenolg^−1^ soil h^−1^)										
		Maize + faba bean		Maize + soybean		Maize + chickpea		Maize + turnip		Average		
		Mono	Inter	Mono	Inter	Mono	Inter	Mono	Inter	Mono	Inter	Mean
2011	0	37.8ab	41.1ab	53.0a	54.1a	28.9b	44.6ab	27.3b	50.9a	36.8a	47.8a	42.2B
	40	32.9c	54.3ab	49.7b	62.7a	34.2c	52.4ab	46.4b	46.0b	40.8b	53.9a	47.3B
	80	60.8ab	56.4ab	76.1a	69.7ab	52.0b	63.4ab	50.4b	51.2b	59.8a	60.2a	60.0A
	**Mean**	**43.8CDE**	**50.6BCD**	**59.6AB**	**62.2A**	**38.4E**	**53.5ABC**	**41.4DE**	**49.4BCD**	**45.8A**	**53.9A**	**49.8**
2012	0	159.7a	153.0a	179.4a	153.4a	145.3a	133.7a	144.9a	132.2a	157.3a	143.1a	150.2A
	40	96.6b	115.1b	120.0b	183.5a	107.6b	121.9b	98.8b	127.0b	105.8b	136.9a	121.3A
	80	134.1ab	137.6ab	173.6a	186.3a	135.5ab	112.0b	134.6ab	150.9ab	144.6a	146.7a	145.6A
	**Mean**	**130.1BC**	**135.2BC**	**157.7AB**	**174.4A**	**129.5BC**	**122.5C**	**126.1BC**	**136.7BC**	**135.8A**	**142.2A**	**139.0**
ANOVA												
	Year (Y)				<0.001						<0.001	
	P rate (P)				0.002						0.077	
	Cropping system (C)				0.000						0.253	
	Y×P				0.006						0.096	
	Y×C				0.400						0.886	
	P×C				0.647						0.253	
	Y×P×C				0.729						0.371	

Values are means of three replicates. Values followed by the same lowercase letters are not significantly different among different cropping systems with the same P rate in one year at the 5% level by LSD (horizonal comparison); values followed by the same capital letters are not significantly different among different P rates (vertical comparison) or among different cropping systems (horizonal comparison) in one year at the 5% level by LSD. Values under ANOVA are the probabilities (*P* values) of the sources of variation.

No effects of fertilizer P on soil nitrate reductase activity were observed in this study in 2011 and 40 kg P ha^−1^ gave lower activities than zero P and 80 kg P ha^−1^ in 2012 (Table S3). Soil nitrate reductase activity was unaffected by intercropping in both years with the exception of maize/faba bean intercropping in 2011 (Table S3). For individual P levels, maize/faba bean and maize/soybean intercropping significantly (P<0.05) enhanced soil nitrate reductase activity by 125% and 199% when receiving 80 kg P ha^−1^ compared with the corresponding monocrops in 2011 (Table S3).

Soil sucrase activity showed no response to fertilizer P rates in either 2011 or 2012 (Table S4). Averaged over the three P rates intercropping did not lead to significant effects on soil sucrase activity compared with the corresponding monocrops across the two years.

## Discussion

### Yield and nutrient acquisition advantages under continuous intercropping

The present study further confirms that maize/faba bean, maize/soybean, maize/chickpea and maize/turnip intercropping systems improve yield and nutrient acquisition for at least 3 to 4 years. For example, results of the first two years of the same field experiment show that averaged over all four intercropping systems there was a 24.3% increase in grain yield in intercropping over the weighted means of the corresponding monocrops [19]. Similar results (about 23.5%) were found in maize/faba bean intercropping on a newly reclaimed desert soil in an arid zone of Ningxia Hui Autonomous Region in northwest China [18]. On a low-phosphorus but high-nitrogen soil over four years in a field experiment the yields of maize grain and faba bean were greater than 43% and 26% in intercropping than in monocropping [20]. Ecological intensification Mozambican maize-based intercropping overyielded by 42.1–88.9% compared to monocropping [9]. Tomato yields increased by 26% in tomato/ryegrass (*Lolium perenne* L.) intercropping compared with sole crops [44] and there was a higher yield in sunflower/soybean intercropping than in corresponding sole crops [45].

Numerous results also indicate that intercropping facilitates both productivity and nutrient acquisition compared with the corresponding monocrops [7], [19], [46]. As a consequence, intercropping significantly removes more nutrients from the soil than monocultures do. For instance, maize/faba bean intercropping showed greater P uptake (by 28–29%) than corresponding monocrops and a 32.9% increase in P uptake by the aboveground parts relative to monocrops [19], [29]. P acquisition of maize/faba bean intercropping increased by 29 and 28% on a relatively fertile soil [17] and by 23.5% on average across three P application rates [18] compared with the corresponding monocultures in a reclaimed desert soil. The present results also confirm that intercropping removes more N in maize-based systems than in monoculture. There are similar results for N acquisition in maize/faba bean, barley/maize [47], and legume/cassava intercropping [48]. It is therefore necessary to know how the soil properties change under such higher yields and greater nutrient removals from soil in intercropping compared with monocultures.

### Soil chemical properties

We found that soil organic matter is less affected by intercropping over a period of three to four years but is dependent on crop combinations. All intercropping involving legumes did not change soil organic matter, but turnip/maize intercropping consistently enhanced the soil organic matter content in both years. The results are not entirely inconsistent with those in agroforestry and grassland ecosystems. Soil carbon concentrations were significantly higher in two tree species combinations than in monoculture sites in Brazil [49]. Different harvest frequencies in poplar short-rotation forestry led to increases in SOC ranging between 9.1 and 30% compared with intensive agricultural systems in a Mediterranean area [35]. In some tree-based intercropping systems, soil organic carbon content was higher than in conventional agricultural fields in Canada [50]. Fornara and Tilman also found that high-diversity grass mixtures stored more soil C than did monocultures [51]. Plant diversity also exerted positive effects on soil carbon storage in a grassland in Germany [52] and in gliricidia (*Gliricidia sepium*)/maize intercropping in Southern Africa [53]. Our results show that the enhanced soil carbon in intercropping is dependent on the crop combinations. This is logical in intercropping with higher nutrient removals from the system compared to natural ecosystems. However, the present results are based on a three-to-four year time scale. Further studies are required over longer time scales.

Intercropping did not reduce soil total N in continuous overyielding and high nitrogen removal from soil for several years. This may be due to the balance of chemical fertilizer N, biological N_2_ fixation by legumes, crop residues and root incorporation into the soil. Firstly, 225 kg N ha^−1^ led to overyielding and stimulated soil N mineralization to some extent. Previous studies have shown that a higher proportion of N in legumes is derived from atmospheric N_2_ fixation in intercrops than in monocrops, 62–82% on average in sandy soil [54]. Similar results were obtained from short-rotation poplar forestry soil with total N increases ranging between 18.1 and 38.9% compared with intensive agricultural soil [35], [55]. Crop residues provided approximately 40 kg ha^−1^ N accumulation in soil which contributed to total N [56], [57], [58]. Intercropping with continuous overyielding still sustained soil total N in our study over four years compared to the corresponding monocrops.

Intercropping reduced soil Olsen P in comparison with the corresponding monocrops and there are several possible explanations for this. P acquisition by intercropping was significantly greater than corresponding monocrops in a newly reclaimed desert soil in northwest China [18] and the first two years of the field experiment used in the present study [19] conducted in a relatively fertile soil. The underlying mechanisms involve the modification of the rhizosphere environment of intercropped species and ultimately benefiting an associated species by increasing P mobilization and utilization of inorganic or organic P in the soil. For example, there is recent evidence that intercropped faba bean roots secrete organic acids or H^+^ ions to increase P mobility and facilitate its uptake by associated intercropped maize roots in maize/faba bean intercropping [20]. Ae et al also found that pigeon pea roots secrete piscidic acid which promotes the release of P from FePO_4_ by chelating iron [26]. Intercropped chickpea may secrete acid phosphatase from its roots to enhance soil organic P mobilization and utilization by associated maize [59]. Intercropped white lupin (*Lupinus albus* L.) increased P uptake via chelation of Ca^2+^ by citrate exuded from roots and subsequently released P from Ca_2_-P complexes [28]. These underlying mechanisms suggest that intercropping exploits various soil P sources by different plant species and lower soil Olsen P.

Soil exchangeable K shows similar trends to soil Olsen-P. This is perhaps not surprising because greater K removals result from overyielding and in our study there was no application of chemical K fertilizer or manure. The reduction in available P or K in the soil can be easily alleviated by application of chemical P or K or manure fertilizer.

Soil CEC is reduced by intercropping in some crop combinations. A previous study has shown that soil CEC is reduced by cultivation and fertilization compared with unmanaged forest land [60] and this is consistent with our present results. Radulov et al. (2011) also reported that CEC is significantly influenced by soil P and K content but not pH or total nitrogen [61]. Therefore, the declines in soil CEC in our present study likely contributed to the decrease in soil available P or K in some cases. Therefore, applications of P or K fertilizers or manures may be suitable practices for alleviating declines in available P and K and also soil CEC under conditions of overyielding and heavy nutrient removal in intercropping systems.

There were no significant differences in soil pH between intercrops and monocrops in the third year (2011) but soil pH was significantly lower in maize/faba bean and maize/turnip intercropping than in the monocrops in the fourth year (2012) of the experiment. Acidification of the rhizosphere and reduction in soil pH have been reported in previous studies on legumes whose roots release large quantities of organic acids or protons, thereby decreasing soil pH [20], [62]. Similar phenomena have been observed by Kumar et al. (1998) in south India where teak (*Tectona grandis* L.)/Leucaena (*Leucaena glauca* L.) intercropping reduced soil pH compared with monocropped teak or Leucaena [63].

### Soil biological properties

Soil urease catalyzes urea hydrolysis to release NH_4_^+^ and enhanced urease activity may increase NH_3_ loss from soils [64], [65]. To some extent urease is an indicator of plant N availability in different cropping systems and environments [66]. In the present work overall soil urease activity was not influenced by different fertilizer P application rates. However, averaged over three P rates, the majority of intercropping did not change the soil urease activities with the exception of maize/soybean intercropping in 2011 and maize/faba bean intercropping in 2012, in which soil urease activity was greater than in the corresponding monocrops. In a greenhouse experiment intercropping of cucumber with onion or garlic increased soil urease activity and the effect lasted several growing seasons compared with monoculture [67]. In contrast, several authors have shown that intercropping increases urease activity due to more efficient N-cycling derived from the soil [67], [68], [69]. Our present results taken together with these previously published results suggest that intercropping has no clear effects on soil urease activity.

Our results demonstrate that intercropping enhances soil acid phosphatase activity compared to monocropping, suggesting that intercropping may utilize more soil organic P which, however, depends on crop combination and P fertilization. Soil extractable P was significantly related to acid phosphatase activity in arid soils [70] because acid phosphatase activity was associated with soil organic P mobilization [71], [72]. Similarly, some studies of peanut/maize intercropping have revealed significantly higher acid phosphatase activity in the intercrop than in monocropped peanut or maize, and P acquisition by intercropping was enhanced substantially in comparison with monocropping [73]. Chickpea and peanut have been identified as species whose roots exudate large amounts of acid phosphatase into their rhizosphere [59], [74] and this may explain the enhanced acid phosphatase activity observed in intercropping systems involving chickpea or turnip.

Nitrate reductase activity is an indicator of available nitrate in soils which influences root nodules and *Rhizobium japonicum* bacteroids [75], [76]. Anaerobic reactions include denitrification and dissimilatory processes and nitrate catalyzes the first step by reducing NO_3_^−^to NO_2_^−^, with NO_2_^−^ further reduced to N_2_O by nitrate reductase. Furthermore, the N_2_O to N_2_ pathway is catalyzed by nitrous oxide reductase [65]. In the present study, averaged over the three P application rates, soil nitrate reductase activity in maize/faba bean intercropping was significantly greater than in corresponding monocrops. This may derive from high N inputs into the soil by enhanced biological N_2_ fixation by faba bean when intercropped with maize [77]. However, the other three intercropping systems did not show similar results to maize/faba bean intercropping, indicating that the effect may be specific to this crop combination.

Sucrase catalyzes the breakdown of water soluble plant material in soils [78]. Sucrase activity is influenced by manure application and application of chemical fertilizers, crop variety, soil pH and temperature [79], [80]. However, our results suggest that three of four years of intercropping did not influence soil sucrase activity.

## Conclusions

This work demonstrates that continuous intercropping for 3–4 years and fertilizer P application enhanced crop productivity and nutrient acquisition compared with the corresponding monocrops. Intercropping did not alter soil OM and maintained soil total N under continuous overyielding and greater nutrient removal in the relatively fertile soil studied. Soil Olsen P, exchangeable K and CEC were more depleted in intercropping than the corresponding monocrops due to greater nutrient removal in intercropping than in the monocultures in a situation where there were no applications of fertilizer K or farmyard manure. Soil Olsen P increased significantly at 40 kg fertilizer P ha^−1^ but did not increase further with additional P to 80 kg P ha^−1^. Therefore, the reduction in Olsen-P, exchangeable K and CEC of soils can be alleviated by proper fertilization. In general, there was no consistent or significant difference in soil pH between intercropping and monocultures. In the majority of cases, soil enzyme activities in intercropping did not differ from the monocrops, with the exception of acid phosphatase activity which was higher in intercropping than the corresponding monocrops. Our investigation further confirms that there are advantages of intercropping in terms of yield and nutrient acquisition, with soil fertility maintained over a period of at least 3–4 years with proper fertilization under conditions of high yields and nutrient removal. Further research is required to examine crop performance over longer time periods and soil properties need to be studied over longer time scales.

## Supporting Information

Table S1 **Soil total N (g kg^−1^) as affected by main effects of P application and subplot effects of cropping system in 2011 and 2012.**
(DOCX)

Table S2 **Soil pH as affected by main effects of P application and subplot effects of cropping system in 2011 and 2012.**
(DOCX)

Table S3 **Nitrate reductase activity as affected by main effects of P application and subplot effects of cropping system in 2011 and 2012.**
(DOCX)

Table S4 **Sucrase activity as affected by main effects of P application and subplot effects of cropping system in 2011 and 2012.**
(DOCX)
