# Supplementary material for: Intercropping Enhances Productivity and Maintains the Most Soil Fertility Properties Relative to Sole Cropping
Source: PLoS One. 2014 Dec 8;9(12):e113984. doi: 10.1371/journal.pone.0113984 (PMC4259307; doi:10.1371/journal.pone.0113984)
Supplement: Table S1 — Soil total N (g kg−1) as affected by main effects of P application and subplot effects of cropping system in 2011 and 2012. (DOCX) [file pone.0113984.s001.docx]

**Table S1** Soil total N (g kg^-1^) as affected by main effects of P application and subplot effects of cropping system in 2011 and 2012.

| Year | P rate (kg ha^-1^) | Soil total N of intercropped and weighted means of corresponding monocropped crops (g kg^-1^) | | | | | | | | | | |
| --- | --- | --- | --- | --- | --- | --- | --- | --- | --- | --- | --- | --- |
|  |  | Maize + faba bean | | Maize + soybean | | Maize + chickpea | | Maize + turnip | | Average | | |
|  |  | Mono | Inter | Mono | Inter | Mono | Inter | Mono | Inter | Mono | Inter | Mean |
| 2011 | 0 | 1.36ab | 1.28b | 1.37ab | 1.25b | 1.35ab | 1.43a | 1.39ab | 1.37ab | 1.37a | 1.33a | 1.35A |
|  | 40 | 1.35ab | 1.35ab | 1.36ab | 1.36ab | 1.31b | 1.32b | 1.35ab | 1.47a | 1.34a | 1.37a | 1.36A |
|  | 80 | 1.32c | 1.43a | 1.30c | 1.35bc | 1.33bc | 1.35abc | 1.36abc | 1.41ab | 1.33b | 1.38a | 1.36A |
|  | **Mean** | **1.34B** | **1.35AB** | **1.34B** | **1.32B** | **1.33B** | **1.37AB** | **1.37AB** | **1.42A** | **1.35A** | **1.36A** | **1.35** |
| 2012 | 0 | 1.22a | 1.23a | 1.20a | 1.20a | 1.20a | 1.22ab | 1.20a | 1.25a | 1.20a | 1.23a | 1.22A |
|  | 40 | 1.25ab | 1.14b | 1.24ab | 1.26a | 1.22ab | 1.18ab | 1.22ab | 1.23ab | 1.23a | 1.20a | 1.22A |
|  | 80 | 1.26a | 1.21a | 1.24ab | 1.28a | 1.20a | 1.24a | 1.27a | 1.23a | 1.24a | 1.24a | 1.24A |
|  | **Mean** | **1.24A** | **1.19B** | **1.23AB** | **1.25A** | **1.21AB** | **1.22AB** | **1.23AB** | **1.24AB** | **1.23A** | **1.22A** | **1.22** |
|  |  |  |  |  |  |  |  |  |  |  |  |  |
| ANOVA |  |  |  |  |  |  |  |  |  |  |  |  |
|  | Year (Y) |  |  |  | ＜0.001 |  |  |  |  |  | ＜0.001 |  |
|  | P rate (P) |  |  |  | 0.016 |  |  |  |  |  | 0.640 |  |
|  | Cropping system (C) |  |  |  | 0.347 |  |  |  |  |  | 0.509 |  |
|  | Y×P |  |  |  | 0.362 |  |  |  |  |  | 0.761 |  |
|  | Y×C |  |  |  | 0.204 |  |  |  |  |  | 0.458 |  |
|  | P×C |  |  |  | 0.253 |  |  |  |  |  | 0.687 |  |
|  | Y×P×C |  |  |  | 0.164 |  |  |  |  |  | 0.147 |  |

Values are means of three replicates. Values followed by the same lowercase letters are not significantly different among different cropping systems with the same P rate in one year at the 5% level by LSD (horizonal comparison); values followed by the same capital letters are not significantly different among different P rates (vertical comparison) or among different cropping systems (horizonal comparison) in one year at the 5% level by LSD. Values under ANOVA are the probabilities (*P* values) of the sources of variation
