# Supplementary material for: Intercropping Enhances Productivity and Maintains the Most Soil Fertility Properties Relative to Sole Cropping
Source: PLoS One. 2014 Dec 8;9(12):e113984. doi: 10.1371/journal.pone.0113984 (PMC4259307; doi:10.1371/journal.pone.0113984)
Supplement: Table S2 — Soil pH as affected by main effects of P application and subplot effects of cropping system in 2011 and 2012. (DOCX) [file pone.0113984.s002.docx]

**Table S2** Soil pH as affected by main effects of P application and subplot effects of cropping system in 2011 and 2012.

| Year | P rate (kg ha^-1^) | Soil pH of intercropped and weighted means of corresponding monocropped crops (2.5:1 v/w) | | | | | | | | | | |
| --- | --- | --- | --- | --- | --- | --- | --- | --- | --- | --- | --- | --- |
|  |  | Maize + faba bean | | Maize + soybean | | Maize + chickpea | | Maize + turnip | | Average | | |
|  |  | Mono | Inter | Mono | Inter | Mono | Inter | Mono | Inter | Mono | Inter | Mean |
| 2011 | 0 | 8.18a | 8.21a | 8.17a | 8.22a | 8.18a | 8.17a | 8.17a | 8.17a | 8.18a | 8.19a | 8.18A |
|  | 40 | 8.16a | 8.18a | 8.15a | 8.24a | 8.15a | 8.17a | 8.19a | 8.15a | 8.16a | 8.19a | 8.17A |
|  | 80 | 8.13a | 8.14a | 8.11a | 8.15a | 8.12a | 8.16a | 8.13a | 8.12a | 8.12a | 8.14a | 8.13B |
|  | **Mean** | **8.15B** | **8.18AB** | **8.15B** | **8.20A** | **8.15B** | **8.17AB** | **8.16AB** | **8.15B** | **8.15A** | **8.17A** | **8.16** |
| 2012 | 0 | 8.13a | 7.90d | 7.98bc | 7.93cd | 7.96cd | 8.04b | 7.93cd | 7.75e | 8.00a | 7.91b | 7.95A |
|  | 40 | 8.10a | 7.82de | 7.98bc | 7.93bcd | 8.02ab | 8.09a | 7.88cde | 7.80e | 7.99a | 7.91a | 7.95A |
|  | 80 | 8.04b | 7.71e | 8.03b | 7.92c | 7.98bc | 8.13a | 7.81d | 7.69e | 7.97a | 7.86b | 7.91B |
|  | **Mean** | **8.09A** | **7.81D** | **8.00B** | **7.93C** | **7.99B** | **8.09A** | **7.87C** | **7.75E** | **7.97A** | **7.89B** | **7.94** |
| ANOVA |  |  |  |  |  |  |  |  |  |  |  |  |
|  | Year (Y) |  |  |  | ＜0.001 | |  |  |  |  | ＜0.001 |  |
|  | P rate (P) |  |  |  | ＜0.001 | |  |  |  |  | 0.002 |  |
|  | Cropping system (C) |  |  |  | ＜0.001 | |  |  |  |  | 0.003 |  |
|  | Y×P |  |  |  | ＜0.001 | |  |  |  |  | 0.892 |  |
|  | Y×C |  |  |  | ＜0.001 | |  |  |  |  | ＜0.001 |  |
|  | P×C |  |  |  | ＜0.001 | |  |  |  |  | 0.839 |  |
|  | Y×P×C |  |  |  | ＜0.001 | |  |  |  |  | 0.892 |  |

Values are means of three replicates. Values followed by the same lowercase letters are not significantly different among different cropping systems with the same P rate in one year at the 5% level by LSD (horizonal comparison); values followed by the same capital letters are not significantly different among different P rates (vertical comparison) or among different cropping systems (horizonal comparison) in one year at the 5% level by LSD. Values under ANOVA are the probabilities (*P* values) of the sources of variation.
