# Supplementary material for: Intercropping Enhances Productivity and Maintains the Most Soil Fertility Properties Relative to Sole Cropping
Source: PLoS One. 2014 Dec 8;9(12):e113984. doi: 10.1371/journal.pone.0113984 (PMC4259307; doi:10.1371/journal.pone.0113984)
Supplement: Table S3 — Nitrate reductase activity as affected by main effects of P application and subplot effects of cropping system in 2011 and 2012. (DOCX) [file pone.0113984.s003.docx]

**Table S3** Nitrate reductase activity as affected by main effects of P application and subplot effects of cropping system in 2011 and 2012.

| Year | P rate (kg ha^-1^) | Nitrate reductase activity of intercropped and weighted means of corresponding monocropped crops (µg NO_2_^-^-N g^-1^ soil d^-1^) | | | | | | | | | | |
| --- | --- | --- | --- | --- | --- | --- | --- | --- | --- | --- | --- | --- |
|  |  | Maize + faba bean | | Maize + soybean | | Maize + chickpea | | Maize + turnip | | Average | | |
|  |  | Mono | Inter | Mono | Inter | Mono | Inter | Mono | Inter | Mono | Inter | Mean |
| 2011 | 0 | 3.391ab | 4.474ab | 2.930ab | 1.085b | 4.331ab | 4.905ab | 4.634ab | 6.073a | 3.822a | 4.134a | 3.978A |
|  | 40 | 4.424a | 4.649a | 2.237a | 4.207a | 3.436a | 4.394a | 4.297a | 3.945a | 3.599a | 4.299a | 3.949A |
|  | 80 | 3.230bcd | 7.271a | 1.772d | 5.304ab | 1.497d | 3.448bcd | 2.546cd | 4.179bc | 2.261b | 5.051a | 3.656A |
|  | **Mean** | **3.682BCD** | **5.465A** | **2.313D** | **3.532BCD** | **3.088CD** | **4.249ABC** | **3.826BCD** | **4.732AB** | **3.227B** | **4.495A** | **3.861** |
| 2012 | 0 | 3.812ab | 3.834ab | 3.745ab | 2.415b | 4.036a | 4.679a | 4.165a | 4.614a | 3.940a | 3.886a | 3.913A |
|  | 40 | 3.352ab | 4.350a | 2.615b | 2.545b | 3.325ab | 3.370ab | 3.861a | 3.890a | 3.288a | 3.539a | 3.414B |
|  | 80 | 3.788abc | 3.829abc | 2.827c | 3.000bc | 3.848abc | 4.003abc | 5.163a | 4.053ab | 3.907a | 3.721a | 3.814A |
|  | **Mean** | **3.651BC** | **4.004AB** | **3.062CD** | **2.653D** | **3.736ABC** | **4.017AB** | **4.396A** | **4.186AB** | **3.711A** | **3.715A** | **3.713** |
| ANOVA |  |  |  |  |  |  |  |  |  |  |  |  |
|  | Year (Y) |  |  |  | 0.630 | |  |  |  |  | 0.690 | |
|  | P rate (P) |  |  |  | 0.688 | |  |  |  |  | 0.771 | |
|  | Cropping system (C) |  |  |  | 0.000 | |  |  |  |  | 0.024 | |
|  | Y×P |  |  |  | 0.403 | |  |  |  |  | 0.534 | |
|  | Y×C |  |  |  | 0.190 | |  |  |  |  | 0.036 | |
|  | P×C |  |  |  | 0.089 | |  |  |  |  | 0.202 | |
|  | Y×P×C |  |  |  | 0.146 | |  |  |  |  | 0.152 | |

Values are means of three replicates. Values followed by the same lowercase letters are not significantly different among different cropping systems with the same P rate in one year at the 5% level by LSD (horizonal comparison); values followed by the same capital letters are not significantly different among different P rates (vertical comparison) or among different cropping systems (horizonal comparison) in one year at the 5% level by LSD. Values under ANOVA are the probabilities (*P* values) of the sources of variation.
