# Supplementary material for: Intercropping Enhances Productivity and Maintains the Most Soil Fertility Properties Relative to Sole Cropping
Source: PLoS One. 2014 Dec 8;9(12):e113984. doi: 10.1371/journal.pone.0113984 (PMC4259307; doi:10.1371/journal.pone.0113984)
Supplement: Table S4 — Sucrase activity as affected by main effects of P application and subplot effects of cropping system in 2011 and 2012. (DOCX) [file pone.0113984.s004.docx]

**Table S4** Sucrase activity as affected by main effects of P application and subplot effects of cropping system in 2011 and 2012.

| Year | P rate (kg ha^-1^) | Sucrase activity of intercropped and weighted means of corresponding monocropped crops (mg glucose g^-1^ soil d^-1^) | | | | | | | | | | |
| --- | --- | --- | --- | --- | --- | --- | --- | --- | --- | --- | --- | --- |
|  |  | Maize + faba bean | | Maize + soybean | | Maize + chickpea | | Maize + turnip | | Average | | |
|  |  | Mono | Inter | Mono | Inter | Mono | Inter | Mono | Inter | Mono | Inter | Mean |
| 2011 | 0 | 40.5ab | 44.2a | 34.6bc | 39.5ab | 31.9c | 38.8abc | 36.9bc | 40.0ab | 36.0a | 40.6a | 38.3A |
|  | 40 | 43.9a | 34.9a | 39.2a | 37.9a | 36.4a | 36.9a | 44.2a | 35.6a | 40.9a | 36.3a | 38.6A |
|  | 80 | 36.5ab | 34.1ab | 31.7b | 32.9ab | 39.3ab | 38.0ab | 39.5ab | 40.8a | 36.8a | 36.5a | 36.6A |
|  | **Mean** | **40.3A** | **37.7AB** | **35.2B** | **36.8AB** | **35.9AB** | **37.9AB** | **40.2A** | **38.8AB** | **37.9A** | **37.8A** | **37.8** |
| 2012 | 0 | 38.6ab | 40.8ab | 36.4b | 37.2b | 39.7ab | 36.8b | 43.8ab | 47.9a | 39.6a | 40.7a | 40.2A |
|  | 40 | 44.9a | 45.1a | 38.5a | 36.7a | 39.1a | 37.8a | 37.6a | 42.3a | 40.0a | 40.5a | 40.3A |
|  | 80 | 43.2ab | 42.6ab | 37.0b | 36.3b | 37.6ab | 41.1ab | 46.0a | 41.7ab | 41.0a | 40.4a | 40.7A |
|  | **Mean** | **42.2ABC** | **42.8AB** | **37.3CD** | **36.7D** | **38.8BCD** | **38.6BCD** | **42.5AB** | **44.0A** | **40.2A** | **40.5A** | **40.4** |
| ANOVA |  |  |  |  |  |  |  |  |  |  |  |  |
|  | Year (Y) |  |  |  | 0.003 | |  |  |  |  | 0.025 | |
|  | P rate (P) |  |  |  | 0.728 | |  |  |  |  | 0.817 | |
|  | Cropping system (C) |  |  |  | 0.002 | |  |  |  |  | 0.909 | |
|  | Y×P |  |  |  | 0.412 | |  |  |  |  | 0.562 | |
|  | Y×C |  |  |  | 0.740 | |  |  |  |  | 0.859 | |
|  | P×C |  |  |  | 0.422 | |  |  |  |  | 0.168 | |
|  | Y×P×C |  |  |  | 0.120 | |  |  |  |  | 0.258 | |

Values are means of three replicates. Values followed by the same lowercase letters are not significantly different among different cropping systems with the same P rate in one year at the 5% level by LSD (horizonal comparison); values followed by the same capital letters are not significantly different among different P rates (vertical comparison) or among different cropping systems (horizonal comparison) in one year at the 5% level by LSD. Values under ANOVA are the probabilities (P values) of the sources of variation.
